# Ib-M1 Antimicrobial Peptide Alters Membrane Permeability and Disrupts *Escherichia coli* O157:H7 Bacillar Morphology

**DOI:** 10.3390/microorganisms14061237

**Published:** 2026-05-30

**Authors:** Mónica Liliana Pérez-Rivera, Ana Elvira Farfán-García, Edgar Javier Rincón-Baron, Johanna Marcela Flórez-Castillo, Oscar Gilberto Gómez-Duarte, Indira Paola Hernández-Peñaranda

**Affiliations:** 1 Facultad de Ciencias Médicas y de la Salud, Instituto de Investigación Masira, Universidad de Santander, Bucaramanga 680003, Colombia; udesliliperez@gmail.com (M.L.P.-R.); afarfan@udes.edu.co (A.E.F.-G.); 2 Corporación de Ciencias Básicas Biomédicas, Universidad de Antioquia, Medellín 050010, Colombia; 3 Facultad de Ciencias Naturales, Universidad de Santander, Bucaramanga 680003, Colombia; ed.rincon@mail.udes.edu.co; 4 Facultad de Ciencias Agrícolas y Veterinarias, Universidad de Santander, Bucaramanga 680003, Colombia; jflorezcas@unimagdalena.edu.co; 5 Division of Pediatric Infectious Diseases and Immunology, Department of Pediatrics, Levine Children’s Hospital, Charlotte, NC 28203, USA; 6 Department of Pediatrics, School of Medicine, Wake Forest University, Charlotte, NC 28203, USA

**Keywords:** bacterial membrane permeability, Ib-M1 antimicrobial peptide, morphological alterations cell surface, *Escherichia coli* O157:H7

## Abstract

The Ib-M1 peptide exhibits bactericidal activity against *Escherichia coli* O157:H7 and low toxicity in mammalian cells. The present study aimed to evaluate the effect of Ib-M1 on *E. coli* O157:H7 membrane permeabilization. For this purpose, the minimum inhibitory concentrations of Ib-M1 for *E. coli* O157:H7 and ML35 were measured. The permeability of the *E. coli* outer and inner membranes was determined by measuring N-phenyl-1-naphthylamine and O-nitrophenyl-β-galactosidase hydrolysis, respectively after bacterial exposure to antimicrobial peptides and control antibiotics. Morphological changes in antimicrobial-exposed *E. coli* O157:H7 were evaluated by scanning electron microscopy following treatment with antimicrobial peptides. Ib-M1 expressed activity against *E. coli* O157:H7 and ML35 at minimal inhibitory concentrations (MIC) of 2.9 ± 1.7 and 6.3 ± 0 μM, respectively. The peptide induced permeabilization of the outer membrane of *E. coli* O157:H7 at all concentrations evaluated and permeabilization of the inner membrane after 50 min at concentrations between 1× MIC and 8× MIC. The morphological changes induced by Ib-M1 led to significant alterations in bacterial shape including collapsed cells and pronounced surface roughness and invaginations. In conclusion, physiological and morphological evidence indicates that the Ib-M1 antimicrobial effect against *E. coli* O157:H7 is mediated by its permeabilizing action on the outer and inner bacterial membranes.

## 1. Introduction

Antimicrobial peptides (AMPs) have attracted significant attention due to their efficacy against a wide spectrum of bacteria and fungi, including antibiotic-resistant strains and those associated with biofilms [1,2]. AMPs exhibit an advantage over antibiotics in that they offer a reduced probability of inducing resistance, due to their preferential attack on the cell membrane and other internal cell targets not typically exploited by conventional antibiotics, such as FTsZ, a GTPase involved in bacterial cell division [3], and DnaK, a heat shock protein associated with the chaperone pathway [4]. Moreover, the swift action of AMPs reduces the opportunity for bacteria to develop repair mechanisms against the alternations or damage caused by antimicrobials, thus hindering the increase in the rate of bacterial mutation [5]. AMPs have been widely studied for the treatment of bacterial infections, and for this purpose, comprehensive investigations have been undertaken on the application of AMPs for bacterial infection treatment, with a focus on reducing toxicity, enhancing resistance to protein degradation, prolonging their half-life in the bloodstream, and achieving profitable mass production [6,7].

Ib-M1 is an analogous peptide derived from Ib-AMP4, with a cationic charge (+6) and an α-helix structure with 20 amino acids [8]. Its in vitro potential has been evaluated in non-pathogenic *E. coli* K-12 (IC_50_: 15 μM) and *E. coli* O157:H7 (MIC: 4.7 μM) [8,9]. Previous reports support the in vitro selective antimicrobial activity of Ib-M1 against *E. coli*, where this peptide exhibits low toxicity toward mammalian cells, demonstrating a 50% cytotoxic concentration (CC_50_) of 395 µM and a selectivity index (SI) of 84.1 in VERO cells, and a CC_50_ of 120 µM and an SI of 9.6 in normal human colon mucosal cells (NCM460) [9,10]. Regarding hemolytic activity, it has not been specifically evaluated for the peptide Ib-M1. Studies conducted with Ib-M6, a peptide belonging to the same family, reported 29% hemolysis at elevated concentrations of 100 µM [8]. Exposure of *E. coli* to Ib-M1 may induce the differential expression of proteins linked to chemotaxis, oxidation–reduction, protection against toxicity from biomolecules, and outer membrane maintenance [10]. Recently, peptides from the Ib-M family have been reported to exert bactericidal effects on non-pathogenic *E. coli* strains through interactions with bacterial membranes [11].

Shiga toxin-producing *E. coli* (STEC, which includes enterohemorrhagic *E. coli* [EHEC]), is an important cause of foodborne illness, affecting approximately 1 million people worldwide, with an annual loss of 13,000 disability-adjusted life years (DALYs) [12,13,14,15]. *E. coli* O157:H7 is the common serotype associated with human infections characterized by watery or bloody diarrhea as well as serious complications including hemorrhagic colitis and hemolytic uremic syndrome [16,17]. Cattle are a major reservoir of *E. coli* O157:H7, but unlike humans, *E. coli* O157:H7 colonization of animal gut is asymptomatic. STEC colonizes the recto-anal junction of cattle and some animals tend to shed *E. coli* O157:H7 more than others; they are called super shedders [18]. Cattle transmit *E. coli* O157:H7 to humans by shedding it in their feces and subsequently contaminating bovine-derived products for human consumption such as meat and dairy products [19] or contaminating water and vegetables, also for human consumption [20,21,22]. While antibiotics are not indicated for the treatment of *E. coli* O157:H7 infection in humans, because they frequently induce HUS, we anticipated that antimicrobial peptides may be used in cattle to decrease cattle *E. coli* O157:H7 colonization and shedding, especially among super shedders, without inducing antimicrobial resistance.

Considering that pathogenic strains such as *E. coli* O157:H7 exhibit differences in cell surface composition, which may influence this interaction, it is essential to evaluate the mechanism of action of Ib-M1 in a pathogenic context. Therefore, in the present study, changes in the membrane integrity of *E. coli* O157:H7 exposed to Ib-M1 were analyzed using membrane permeability assays and morphostructural studies to broaden and complement previously reported findings.

## 2. Materials and Methods

### 2.1. Ib-M1 Peptide

Ib-M1 was synthesized by Biomatik^®^ (Kitchener, Canada) based on the peptide sequence described in Table 1. The peptide purity was 98.6% by HPLC analysis, according to the certificate of analysis provided by the supplier. The general characteristics of Ib-M1 have been previously described by Flórez-Castillo [8]. Ib-M1 stock solutions were prepared by dissolving the peptide in 10 mM Tris-HCl buffer (pH 7.4) at a concentration of 2 mM. Subsequently, aliquots of 70 µL were dispensed into Eppendorf vials and stored at −80 °C for no more than 7 months.

### 2.2. Antibiotics

The alterations induced by Ib-M1 on the *E. coli* cell membrane were compared with two reference antibiotics selected for their well-documented mechanisms of action. Polymyxin B was chosen as a positive control because it alters the membrane integrity of gram-negative bacteria [23,24]. Streptomycin was chosen as a negative control because it disrupts intracellular processes by binding to the 30S ribosomal subunit, which ultimately leads to inhibition of protein synthesis in bacteria, without affecting bacterial membrane integrity [25].

### 2.3. Strains

Two strains of *E. coli* were used in this study: *E. coli* O157:H7 (Migula) Castellani and Chalmers (ATCC^®^ 43888™) and *E. coli* ML35 (Migula) Castellani and Chalmers (ATCC^®^ 43827™). These strains were kept cryopreserved at −80 °C in Luria–Bertani (LB) broth with 15% glycerol. Before each experiment, a subculture of *E. coli* was grown in 5 mL of LB broth and incubated at 37 °C for 18 to 20 h. All experiments were carried out using *E. coli* O157:H7, except for the inner membrane permeability assay, which was conducted with *E. coli* ML35. This latter strain is characterized by its constitutive expression of cytoplasmic β-galactosidase and the absence of the Lac permease transporter, which makes it suitable for use in combination with easily hydrolyzable enzymatic substrates such as ONPG (O-nitrophenyl β-galactosidase) [26].

### 2.4. Minimum Inhibitory Concentration

The minimum inhibitory concentration (MIC) was determined for *E. coli* O157:H7 and *E. coli* ML35. The inhibitory activity of Ib-M1 against *E. coli* was evaluated at two cellular densities: 5 × 10^5^ CFU/mL and 1 × 10^8^ CFU/mL. The former concentration was selected based on adherence to the standard concentration recommended by the Clinical and Laboratory Standards Institute (CLSI) for evaluating antibacterial effectiveness. The latter concentration was chosen because it has been utilized in previous studies involving scanning electron microscopy, which in this work was evaluated only in *E. coli* O157:H7. In contrast, *E. coli* ML35 was used exclusively for the inner membrane permeability assay [27,28]. The MICs of the antimicrobials were determined using the microdilution method, as described in protocol M07-A9 of the CLSI [29]. Subcultures of *E. coli* were used to achieve final concentrations of 1 × 10^8^ CFU/mL and 5 × 10^5^ CFU/mL by spectrophotometry at OD_600_. Serial dilutions (1:2) of antimicrobials were prepared in 96-well round-bottom plates with Müeller–Hinton (MH) broth to yield final concentrations of Ib-M1 (200 to 6.25 µM), streptomycin (200 to 6.25 µM), and polymyxin B (16.4 to 0.128 µM). Next, 100 µL of *E. coli* was added to each well, followed by incubation at 37 °C for 22 h. Negative controls were implemented by adding MH broth to the wells and growth controls used MH broth and *E. coli*. At the end of the incubation period, the MIC was determined as the lowest concentration of the evaluated compound that inhibited the visible growth of *E. coli* among the tested wells.

### 2.5. Outer Membrane Permeability Assay

A fluorescent NPN (N-phenyl naphthylamine) probe was used to evaluate the integrity of the *E. coli* O157:H7 outer membrane. The fluorescence of the NPN probe increases upon transition from a hydrophilic to a hydrophobic environment. In this context, if the tested antimicrobials destabilized the outer membrane of the *E. coli* O157:H7, the probe would bind to the hydrophobic molecules exposed on the outer membrane, resulting in changes to the emission of fluorescence measurable through spectrofluorometry [30].

*E. coli* O157:H7 was cultivated on LB agar for 18 h at 37 °C. The colonies were then resuspended in 5 mM HEPES and centrifuged at 4500 rpm for 5 min, with the supernatant being discarded. This procedure was done twice. In the final wash, *E. coli* O157:H7 was adjusted to an absorbance of 0.5 at a wavelength of OD_600_. Thereafter, it was centrifuged again; the supernatant was discarded; and the pellet was resuspended in HEPES at half of the initial volume. Afterward, black flat-bottom 96-well plates were used to dispense 50 µL of antimicrobials at final concentrations of 1× MIC, 2× MIC, 4× MIC, 8× MIC, 16× MIC, and 32× MIC. Subsequently, 50 µL of 160 µM NPN was added, followed by 100 µL of the *E. coli* O157:H7 bacterial suspension. Finally, spectrofluorometric readings were performed at an excitation wavelength of 355 nm and an emission wavelength of OD_405_. The fluorescence was recorded as a function of time until no further increase in the signal was observed. Various control groups were used to verify that the observed fluorescence corresponded to the binding of NPN to phospholipids of the outer membrane. The following control wells were used: a. HEPES, b. HEPES + NPN, c. HEPES + *E. coli*, d. HEPES + NPN + *E. coli* [31].

### 2.6. Inner Membrane Permeability Assay

O-nitrophenyl-β-galactosidase (ONPG) was used to detect the damage caused by Ib-M1 to the inner membrane of *E. coli*. ONPG is a substrate that reacts with the β-galactosidase found in the periplasmic space of permeabilized bacteria, producing orthonitrophenol, a yellow chromogen that can be quantified by spectrophotometry [26]. For this purpose, *E. coli* ML35 was selected as it lacks the permease enzyme, which permits verification that the interaction with ONPG occurred specifically due to alterations to the inner membrane effected by the compounds of interest.

*E. coli* ML35 was grown in 5 mL of LB broth and incubated at 37 °C for 20 h. The microorganisms were then suspended in phosphate-buffered saline (PBS) pH 7.4 with a turbidity equivalent to 2 on the McFarland scale. Two additional washes were performed, and the solution was centrifuged at 4500 rpm for 10 min. The supernatant was discarded, and the pellet was resuspended in PBS. The bacterial suspension was adjusted to 5 × 10^5^ CFU/mL based on a standard bacterial growth curve of CFU/mL and OD_600_ for spectrophotometric analysis. Next, 50 µL of Ib-M1, polymyxin B, or streptomycin was added to 96-well plates at final concentrations of 1× MIC, 2× MIC, 4× MIC, 8× MIC, and 16× MIC; after which, 50 µL of ONPG at 6 mM and 100 µL of *E. coli* ML35 bacterial suspension were added. Finally, spectrophotometric reading was carried out using a Multiskan Sky spectrophotometer at a wavelength of OD_415_ every 20 min for 5 h. *E. coli* + ONPG and *E. coli* + PBS were used as controls [32]. The results of the experiment were expressed in terms of the absorbances obtained at an array of exposure times to the compounds. Alterations in the permeability of the *E. coli* ML35 inner membrane were indicated by increased absorbance values due to the reaction between the ONPG and the β-galactosidase enzyme released into the extracellular medium.

### 2.7. Scanning Electron Microscopy

A bacterial suspension in 5 mL of PBS at a concentration of 1 × 10^8^ CFU/mL was treated with 1× MIC of Ib-M1, polymyxin B, or streptomycin for 30, 60, or 120 min at 37 °C. After each period, the samples were centrifuged at 4000 rpm for 5 min, followed by three washes with 1 mL of PBS 0.1 M and fixation with 200 µL of 4% glutaraldehyde overnight at 4 °C. The following day, two additional washes with 0.1 M PBS were carried out, and the samples were post-fixed with 1% osmium tetroxide for one hour at room temperature, followed by three final washes with distilled water. Subsequently, the samples were dehydrated by incubation in a gradual ethanol series with changes every 25 min, until a final concentration of 100% ethanol was achieved. Next, 100 µL of a 1:1 mixture of hexamethyldisilazane (HMDS):absolute ethanol was added to the samples and incubated for 1 h, followed by a final addition of HMDS and overnight incubation in a desiccator [33]. The samples were set onto conductive double-sided carbon tape and coated with gold–palladium using a Denton Vacuum Desk IV ionizer (Moorestown, NJ, USA) for 10 min. Observations and microphotographic recordings were made using a JEOL JSM-6490LV scanning electron microscope (Peabody, MA USA).

## 3. Results

### 3.1. Ib-M1 Expresses Antimicrobial Activity at Different E. coli Cell Densities 

Ib-M1 demonstrated inhibitory activity against *E. coli* O157:H7 at concentrations of 2.9 ± 1.7 µM and 100 ± 0 µM for cellular densities of 5 × 10^5^ CFU/mL and 1 × 10^8^ CFU/mL, respectively. For the reference antibiotics, the MICs of the polymyxin B and streptomycin were 0.06250 ± 0.0 µM and 4.7 ± 1.6 µM at a final concentration of 5 × 10^5^ CFU/mL and 0.8 ± 0.0 µM and 100 ± 0.0 µM for 1 × 10^8^ CFU/mL, respectively (Table 2). Antimicrobial activity was also evaluated for the *E. coli* ML35 strain at a concentration of 5 × 10^5^ CFU/mL. The MICs obtained are shown in Table 2.

### 3.2. Outer Membrane Permeability of E. coli Exposed to Ib-M1

**Figure 1:**
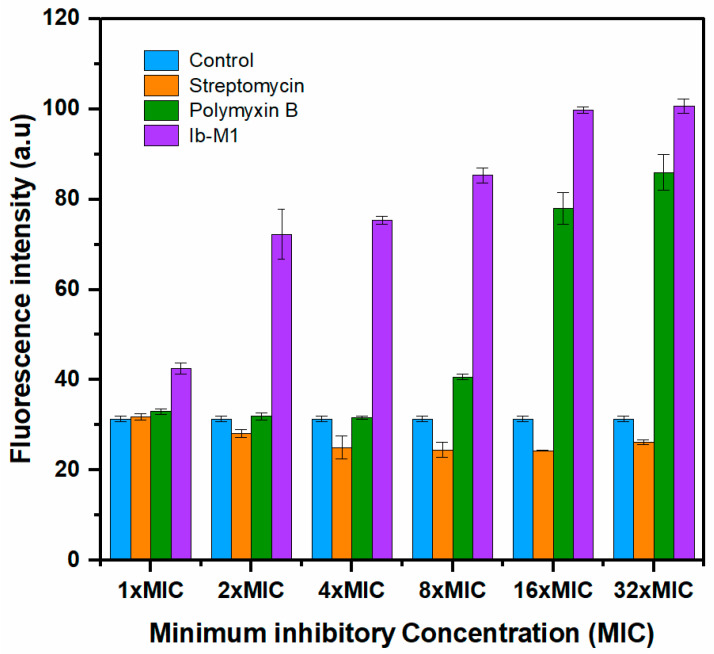
Permeabilization of the *E. coli* O157:H7 outer membrane. Each concentration of Ib-M1, polymyxin B and streptomycin was evaluated in triplicate. Results are expressed in terms of the arithmetic average ± standard deviation of fluorescence intensity. Arbitrary units (a. u.) indicate the relative intensity of the fluorescent signal from the NPN bound to hydrophobic molecules in the outer membrane. The data is a representation of two independent experiments with similar results. Control: *E. coli* O157:H7 + NPN.

Ib-M1 permeabilized the outer membrane of *E. coli* O157:H7 at all concentrations tested. However, no substantial increase in fluorescence was observed beyond 16× MIC (Figure 1). In the case of polymyxin B, the permeabilizing effect was observed from 8× MIC, while concentrations lower than 4× MIC exhibited emissions close to those of the control group. All streptomycin concentrations exhibited fluorescence emissions similar to those of the *E. coli* growth control (Figure 1).

### 3.3. Inner Membrane Permeability of E. coli Exposed to Ib-M1

**Figure 2:**
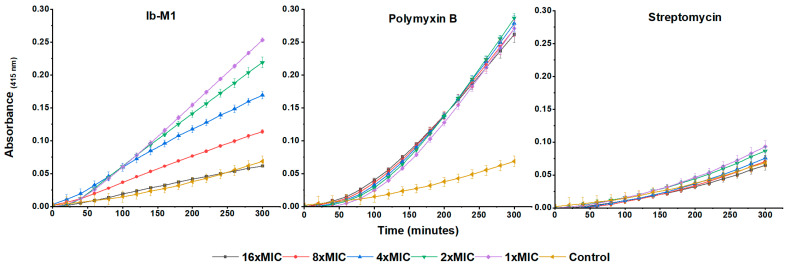
Inner membrane permeabilization of *E. coli* ML35. Each concentration was evaluated in triplicate, and the results are expressed in terms of the arithmetic average ± standard deviation of absorbances obtained at 405 nm. The absorbances indicate the production of ortho-nitrophenol resulting from the hydrolysis of ONPG catalyzed by the enzyme β-galactosidase. The data is a representation of two independent experiments with similar results (Figure S2 and Table S1). Control: *E. coli* ML35 + ONPG.

Ib-M1 affected the permeability of the *E. coli* ML35 inner membrane (Figure 2). The findings showed that the chromogenic reaction increased after 50 min at concentrations of 1× MIC, 2× MIC, 4× MIC, and 8× MIC, with 1× MIC causing the greatest membrane permeability. However, the 16xMIC concentration exhibited similar behavior to the growth control. With respect to the reference antibiotics, polymyxin B caused permeabilization of the inner membrane after 110 min of exposure at all concentrations evaluated (Figure 2), whereas no effect was observed for streptomycin on the permeability of the *E. coli* ML35 inner membrane (Figure 2).

### 3.4. Morphological Alterations of E. coli Exposed to Ib-M1

**Figure 3:**
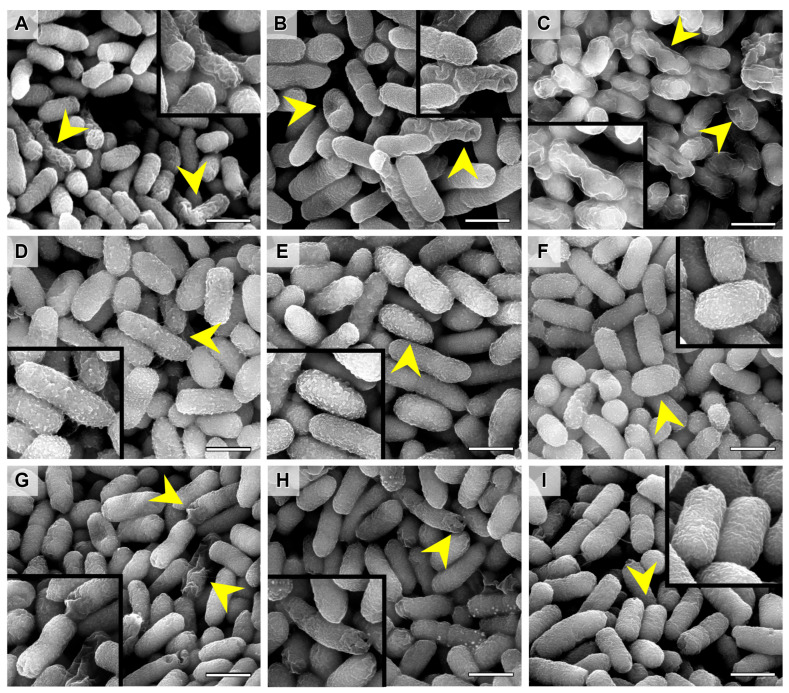
Morphological alterations of *E. coli* O157:H7 treated with Ib-M1, polymyxin B and streptomycin. (**A**) *E. coli* O157:H7 exposed to Ib-M1 for 30, (**B**) 60 and (**C**) 120 min; (**D**) polymyxin B for 30, (**E**) 60 and (**F**) 120 min; (**G**) streptomycin for 60 and (**H**) 120 min; and (**I**) untreated *E. coli* O157:H7 after 120 min. The arrows indicate the morphological alterations observed on the cell surface of *E. coli* O157:H7 after exposure to antimicrobials. The scale bar incorporated into micrographs is equivalent to a length of 1 μm for all cases. The image of each micrograph was enlarged in the upper right corner or the lower left corner.

Changes in the bacillary shape and notable invaginations were observed on the surface of *E. coli* O157:H7 treated with Ib-M1 during the first 60 min (Figure 3B). These lesions were more evident after 120 min, with more-pronounced changes in the bacterial cell and shadowed areas on the cell surface, because of the partial loss of bacterial density (Figure 3C and Figure S3).

*E. coli* O157:H7 cells treated with polymyxin B exhibited multiple blisters on their surface. These lesions were observed in most of the bacteria and at each evaluation time (Figure 3D–F). When *E. coli* O157:H7 was treated with streptomycin, it displayed clefts, invaginations, and roughness in a small number of bacteria between 60 and 120 min; however, a large part of the bacterial population retained its bacillary shape, without lesions on its surface (Figure 3G,H). In untreated *E. coli* O157:H7, the bacteria presented a complete bacillary shape, without surface lesions or cellular debris (Figure 3I).

## 4. Discussion

This study evaluated the bactericidal effects of the Ib-M1 peptide on *E. coli* O157:H7 and investigated its mechanism of action on both the outer and inner membranes of *E. coli.* Ib-M1 induced membrane disruption in *E. coli* O157:H7. Evidence from in vitro permeability tests and electron microscopy demonstrated that lesions on the cellular surface ultimately lead to bacterial cell death. These findings, combined with the fact that these peptides exhibit minimal eukaryotic cytotoxicity, suggest their potential as an alternative to antibiotics for managing infections in mammalian hosts.

Before evaluating the membrane integrity, the MIC of the Ib-M1 peptide in *E. coli* ML35 was determined for use in the inner membrane permeability assays. It was observed that *E. coli* ML35 was less sensitive to Ib-M1 and polymyxin B compared with *E. coli* O157:H7. This variance in antimicrobial activity among different strains may be linked to their responses to growth, survival, or adaptation conditions, as previously discussed in another study [9].

The MICs were also determined at two distinct cellular densities. It was noted that, when using a cell density of 1 × 10^8^ CFU/mL, the Ib-M1 MIC was 34.5 times higher than that found for 5 × 10^5^ CFU/mL. Similarly, the required concentrations for streptomycin and polymyxin B were 12.8 and 21.3 times higher, respectively. The relationship between the concentration of an antimicrobial and cell density is a factor that influences both bacterial morphology and antimicrobial activity of the studied microorganism [27,34]. Cellular density may affect the performance of antimicrobials, comparable to the “inoculum effect”, which is defined as an eightfold-or-greater increase in the MIC when using an inoculum 100 times higher than that recommended by the CLSI [28]. Peptides such as melittin, indolicin, and LL-37 have demonstrated MIC increases of between 3- and 100-fold when the inoculant concentration is increased from 5 × 10^5^ to 1 × 10^8^ CFU/mL. Similarly, this behavior is paralleled by that of conventional antibiotics such as ciprofloxacin and gentamicin [28,35].

The role of positively charged amino acid residues has been implicated in the electrostatic attraction phase between AMP and the bacterial membrane. The presence of seven arginine residues confers a cationic charge on the Ib-M1 peptide, which could promote outer membrane permeabilization [11] as well as drive its antimicrobial effectiveness against *E. coli* O157:H7 [8,9]. The ability of arginine to disrupt the bacterial cell membrane has been attributed to the presence of the guanidinium group, which forms hydrogen bonds with the phosphate groups of membrane phospholipids [36,37]. Another amino acid that may promote the insertion of Ib-M1 into the membrane is tryptophan; this amino acid can associate with the interfacial region of lipid bilayers due to the presence of the indole functional group in its side chain, which interferes with the hydrophobic interactions between the acyl chains of the lipids [38,39].

Regarding the assays performed on the *E. coli* ML35 inner membrane, the results indicated that higher concentrations of Ib-M1 (8× MIC (50.4 μM) and 16× MIC (100.8 μM)) induced lower permeability of the inner membrane. Although this behavior would not typically be expected, it has been previously observed in peptides, such as cecropin, wherein the permeability of the inner membrane was dependent on the concentration of the peptide until the MIC was reached. Thereafter, regardless of the concentration used, no changes were observed in the permeability of the inner membrane [40].

A possible explanation for the behavior of Ib-M1 in the permeabilization of the inner membrane could be related to that observed for the native peptide Ib-AMP4. In the case of Ib-AMP4, a decrease in liposomal calcein leakage was observed at concentrations greater than 100 μg/mL (40 μM) of Ib-AMP4. The authors attributed this behavior to the possible membrane saturation due to the insertion of the peptide, which leads to an increase in lateral pressure and causes the membrane to rupture. Thereafter, the membrane deformation caused by the insertion of the peptide gradually diminished owing to mass exchange and promoted the recovery of the membrane. This, in turn, affects the flexibility of the membrane, which could explain its resistance to peptide insertion at higher concentrations [41]. Another possibility is that AMP may inhibit β-galactosidase activity. For example, molecular dynamics simulations have identified short peptide motifs that have this effect [42]. However, it is important to note that, in various studies using ONPG to evaluate the effect of cationic antimicrobial peptides on the inner membrane of *E. coli* ML35, an increase in permeation was observed depending on the concentration evaluated [43,44].

Morphological alterations observed in *E. coli* O157:H7 complemented the findings of the membrane permeability assay. Ib-M1 induced changes on the cell surface, in the form of roughness and collapsed cells. These lesions were more pronounced and were observed in a larger proportion of bacteria after 120 min of exposure to the peptide. At none of the evaluated times was Ib-M1 observed to cause blisters like those produced by polymyxin B or other lytic-action AMPs such as magainin and melittin [34]. Collapsed cells and deep roughness were observed when streptomycin and Ib-M1 were used, with the most pronounced effects observed in *E. coli* O157:H7 exposed to Ib-M1.

Morphological changes similar to those observed following exposure to Ib-M1 have also been reported for *E. coli* exposed to peptides such as Temporin L. In the referenced study, in addition to demonstrating an increase in *E. coli* membrane permeability, it was reported that the perturbation of the organization of the lipid bilayer did not induce bacterial lysis. Instead, it resulted in the formation of “ghostlike” cells characterized by a translucent and empty appearance, but, nevertheless, even dead cells maintained their shape and DNA content [45].

## 5. Conclusions

The results of this study demonstrate that Ib-M1 disrupts the integrity of the *E. coli* O157:H7 cell membrane, suggesting its potential antimicrobial activity targeting bacterial membrane structures. Ib-M1 induced permeabilization of the *E. coli* ML35 inner membrane and the *E. coli* O157:H7 outer membrane. The peptide also induced morphological changes on the surface of *E. coli* O157:H7, leading to increased roughness and invaginations, thereby modifying the bacterial bacillar shape. Further research is necessary to explore whether Ib-M1 interacts with intracellular bacterial components that contribute to its antimicrobial action. Undertaking such investigations would significantly enhance our understanding of the Ib-M1 antimicrobial peptide mechanisms of action.

We anticipate that Ib-M1 peptides may have a role in the elimination of *E. coli* O157:H7 shedding from cattle’s gut and, by decreasing food product contamination, they may be effective in the prevention *E. coli* O157:H7 disease outbreaks.

## Figures and Tables

**Table 1:** Amino acid sequence of the Ib-M1 peptide.

Position		1	2	3	4	5	6	7	8	9	10	11	12	13	14	15	16	17	18	19	20	
Sequence	H_2_N-	E	W	G	R	R	M	M	G	R	G	P	G	R	R	M	M	R	W	W	R	-CONH_2_

E: glutamic acid; W: tryptophan; G: glycine; R: arginine; M: methionine; P: proline, H_2_N-: N terminal; -CONH_2_: amidated C terminal.

**Table 2:** Antimicrobial activity of Ib-M1 at different cell densities of *E. coli.*

*E. coli* Strain	Cell Density (CFU/mL)	MIC (µM ± S.D.)		
		Ib-M1	Polymyxin B	Streptomycin
O157:H7	5 × 10^5^	2.9 ± 1.7	0.0625 ± 0.0	4.7 ± 1.6
	1 × 10^8^	100 ± 0.0	0.8 ± 0.0	100 ± 0.0
ML35	5 × 10^5^	6.3 ± 0.0	0.1 ± 0.0	6.3 ± 0.0

Each concentration was evaluated in triplicate in two independent experiments. The results are expressed in terms of the arithmetic average of each group ± standard deviation (S.D).

## Data Availability

The original contributions presented in this study are included in the article/Supplementary Materials. Further inquiries can be directed to the corresponding author.

## Supplementary Materials

The following supporting information can be downloaded at https://www.mdpi.com/article/10.3390/microorganisms14061237/s1: Figure S1: Numerical values used to build the figure for OM permeability, https://doi.org/10.5281/zenodo.19154200; Figure S2: Numerical values used to build the figure for IM permeability, https://doi.org/10.5281/zenodo.19154493; Table S1: Numerical values of minimum inhibitory concentration, https://doi.org/10.5281/zenodo.19154635; Figure S3: Raw images of morphological alterations of *E. coli* O157:H7 treated with Ib-M1, polymyxin B and streptomycin, https://doi.org/10.5281/zenodo.19154574.

## Abbreviations

The following abbreviations are used in this manuscript:

AMPs	Antimicrobial peptides
FTsZ	Filamentous temperature-sensitive mutant Z
GTPase	Guanosine triphosphate hydrolase enzyme
DnaK	Chaperone protein DnaK
DALYs	Disability-adjusted life years
HUS	Hemolytic uremic syndrome
HPLC	High-performance liquid chromatography
ATCC	American Type Culture Collection
LB	Luria–Bertani
ONPG	O-nitrophenyl β-galactosidase
MIC	Minimum inhibitory concentration
CLSI	Clinical and Laboratory Standards Institute
CFU	Colony forming unit
MH	Müeller–Hinton
NPN	N-phenyl naphthylamine
rpm	Revolution per minute
HEPES	4-(2-Hydroxyethyl)-1-piperazineethanesulfonic acid
PBS	Phosphate-buffered saline
HMDS	Hexamethyldisilazane
